# A mouse model of corneal endothelial decompensation using cryoinjury

**Published:** 2013-06-05

**Authors:** Sang Beom Han, Hengpei Ang, Deepa Balehosur, Gary Peh, Shyam S. Chaurasia, Donald T. H. Tan, Jodhbir S. Mehta

**Affiliations:** 1 Singapore National Eye Centre, Singapore; 2 Tissue Engineering and Stem Cell Group, Singapore Eye Research Institute, Singapore; 3 Department of Ophthalmology, Sungkyunkwan University School of Medicine, Samsung Medical Center, Seoul, Korea; 4 SRP Neuroscience and Behavioral Disorders, Duke-NUS Graduate Medical School, Singapore; 5 Department of Ophthalmology, Yong Loo Lin School of Medicine, National University of Singapore, Singapore; 6 Department of Clinical Sciences, Duke-NUS Graduate Medical School, Singapore

## Abstract

**Purpose:**

To develop a mouse model of bullous keratoplasty and evaluate the safety and efficacy of cryoinjury-induced corneal endothelial decompensation.

**Methods:**

Transcorneal freezing was performed on the right eye of each mouse. One cycle of cryoinjury was performed in 18 eyes (group A), and three cycles were performed in 17 eyes (group B). Pachymetry and intraocular pressure (IOP) measurements were done preoperatively, as well as at 1, 3, 7, 14, and 21 days after cryoinjury. At each post-cryoinjury time point, three mice from each group were euthanized, and the corneas underwent histology and electron microscopy.

**Results:**

In both groups, significant corneal edema was noted at post-cryoinjury day 1, which was maintained throughout the study period. IOP remained within normal range in group A, but increased significantly with time in group B (p=0.011 at day 1, 0.038 at day 3, 0.026 at day 14, and 0.008 at day 21). In group B, serious complications including hyphema (one case), severe iridocorneal adhesion (15 cases), and total cataract (three cases) were detected, while only mild iridocorneal adhesion (four cases) and cataract (three cases) were noted in group A. Live/dead cell assay, hematoxylin and eosin staining, and scanning electron microscopy revealed successful ablation of corneal endothelial cells and absence of regeneration in both groups. Hematoxylin and eosin staining and terminal deoxynucleotidyl transferase-mediated nick end labeling assay showed that apoptosis was mainly confined to the posterior stroma and endothelium in group A, while severe apoptosis was observed throughout all layers of the cornea in group B.

**Conclusions:**

One cycle of cryoinjury was safer than three, while both were equally effective in inducing bullous keratopathy. This cryoinjury mouse model of bullous keratopathy was a consistently reproducible model that can be used for further studies on endothelial cell damage and rescue therapy.

## Introduction

Various animal models of corneal endothelial decompensation have been described in the literature [1-7], for example, mechanical injury [2-5], that is, scoring of Descemet’s membrane (DM) [2,5], intracameral injection of magnetic foreign particles [6], or chemical approaches using intracameral injection of toxic chemicals [1,7], for example, benzalkonium chloride [7]. Although these methods have been shown to be effective in destroying mouse corneal endothelial cells (CECs), these methods are time-consuming, inconsistently reproducible, and technically difficult, and have a risk of marked anterior chamber (AC) inflammation and damage to intraocular structures, thus precluding widespread use by researchers [7].

Since Maumenee et al. [8] first described the destruction of the CEC layer by transcorneal freezing, cryoinjury has been a popular method of inducing bullous keratopathy in animal models, including the rabbit, rat, cat, and monkey, because of its simple, effective, and non-invasive nature [1,9-16]. However, the use of cryoinjury in a mouse model has never been reported. Previously described models of bullous keratopathy in mouse eyes used intracameral injection of toxic agents or mechanical scraping of the CEC layer [7,17]. For facilitation of research on corneal endothelial disease, a simple and easy method for inducing corneal endothelial decompensation in mice is needed. In the present study, we evaluated the efficacy and safety of transcorneal freezing in inducing corneal endothelial decompensation in the mouse eye and optimized the dose of the cryoinjury to establish a mouse model of bullous keratopathy.

## Methods

### Animals

All animal experiments were performed according to the care of experimental animal guidelines, and all protocols were approved by the Institutional Animal Care and Use Committee of SingHealth, Singapore. All animals were treated according to tenets of the Association for Research in Vision and Ophthalmology’s statement for the Use of Animals in Ophthalmic and Vision Research. C57BL/6 mice were bred and maintained at the SingHealth Experimental Medical Centre (Singapore General Hospital, Singapore).

### Cryoinjury treatment

**Figure 1:**
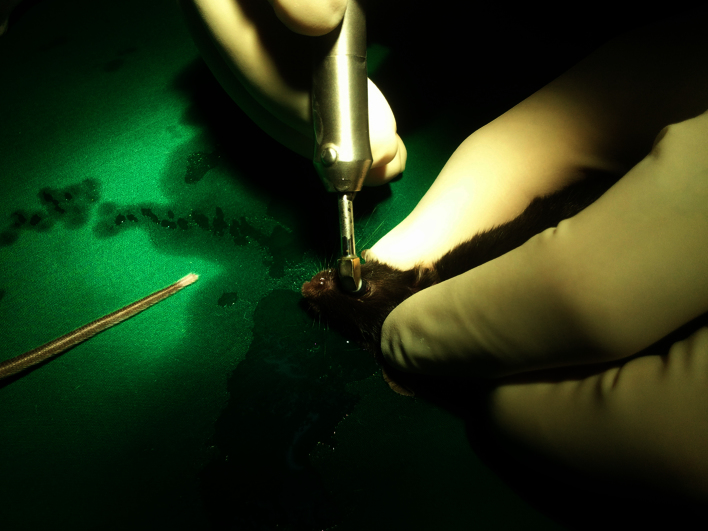
Transcorneal freezing using a cryoprobe with a 2.5 mm diameter on the right eye of a mouse.

A total of 35 mice were randomly divided into two groups, and one (group A, n=18) and three (group B, n=17) cycles of cryoinjury were applied to the right eye of each mouse. Briefly, transcorneal freezing was initiated by gently placing a cryoprobe made of stainless steel (2.5 mm in diameter; flat tip; ERBE Elektromedizin GmbH, Tübingen, Germany), precooled to −80 °C, on the central cornea (Figure 1). A cryoprobe with a diameter of 2.5 mm was chosen as it is similar to the corneal diameter (2.6 mm) of C57BL/6 mice [18]. No pressure was applied to avoid damage to adjacent tissue including the lens and the trabecular meshwork. The lateral surface of the probe, instead of the tip, was placed on the cornea to maximize the contact surface. The cryoprobe was kept on the corneal surface until an ice ball formed on the cornea and covered the entire corneal surface, which corresponds to the duration of 3 s, as the defect on the endothelium was shown to be the same size as the ice ball [1]. Immediately after freezing, the cryoprobe was freed from the corneal surface with irrigation with a balanced salt solution, and the cornea was allowed to thaw spontaneously. In group B, the cryoprobe was applied three times as described above, but with a 1 min interval between each application. No topical medication was applied during the study period.

### Anterior segment examination and Intraocular pressure measurements

The cryoinjured mice were followed for 21 days. Preoperatively and at 1, 3, 7, 14, and 21 days after the cryoinjury, examinations including anterior segment optical coherence tomography (AS-OCT), tonometry, and slit-lamp photography were performed. Central corneal thickness (CCT) was measured using AS-OCT (RT-vue, Optovue, Fremont, CA). Intraocular pressure (IOP) was measured using a rebound tonometer (Tonolab Rebound Tonometer, Icare Finland Oy, Helsinki, Finland). The CCT and IOP measurement were performed three times in each eye, and the average of the three readings was taken at each time point. Slit-lamp photography was performed with a Nikon FS-3V Zoom Photo Slit Lamp (Nikon, Tokyo, Japan) or a Micron III (Phoenix Research Laboratories, Pleasanton, CA).

### Histologic examination

At each observation time point (1, 3, 7, 14, and 21 days after the cryoinjury), three mice from each group were euthanized, and the eyes that underwent cryoinjury were enucleated for further evaluations. Under deep anesthesia with intraperitoneal injection of ketamine hydrochloride (50- 75 mg/kg) and Xylazil (5- 8 mg/kg), mice were euthanized using intraperitoneal overdose anesthesia with sodium pentobarbital (60- 150 mg/kg).

Of the three eyes from each group at each time point, two eyes were used for histological examinations. The corneas were excised and bisected. One half of each cornea was used to evaluate CECs using a live/dead cell assay as previously described [19]. Briefly, corneas were placed endothelial side up and 0.25% trypan blue was applied dropwise for 90 s. Alizarin red S (0.20%; pH 4.2) was then applied for 90 s. The corneas were immersed in a glutaraldehyde fixative solution (2.48%; osmolality 301 mOsm/kg, pH 7.2) for 10 min. All cornea samples were examined using an inverted light microscope (Eclipse Ti Nikon, Tokyo, Japan). The other half of each cornea was embedded in optimal cutting temperature (OCT) compound (Leica Microsystems, Nussloch, Germany). The tissue was stored at −80 °C until sectioning. Sagittal 6 μm sections were serially cut using a Microm HM550 cryostat (Microm, Walldorf, Germany). The sections were placed on polylysine-coated glass slides (Thermo Scientific, Waltham, MA) for hematoxylin and eosin (H&E) staining and terminal deoxynucleotidyl transferase-mediated nick end labeling (TUNEL) assay. The H&E-stained slides were observed under a light microscope (Eclipse Ti, Nikon, Tokyo, Japan). TUNEL assay was performed using the In Situ Cell Death Detection Kit, TMR Red (Roche, Mannhein, Germany), according to the manufacturer’s protocol, and the slides were examined under a fluorescent microscope (Zeiss Axioplan 2, Carl Zeiss, Oberkochen, Germany). The mean numbers of apoptotic cells in the TUNEL samples were calculated by counting the number of cells in five non-overlapping areas (0.1 mm × 0.1 mm) of three separate corneal sections, separated from each other at least by 0.2 mm to avoid overlap.

### Scanning electron microscopy

To confirm the changes in the corneal endothelium, we performed scanning electron microscopy (SEM) using one cornea from each group at each time point. The globes were immersed in a fixative solution, containing 2.5% glutaraldehyde in 0.1M sodium cacodylate (pH 7.4; Electron Microscopy Sciences, Hatfield, PA) overnight at 4 °C. The corneas excised from the globes were washed three times in distilled water for 10 min each, and were kept in 1% osmium tetroxide (FMB, Singapore) at 22 °C for 2 h for final fixation. The corneas were then dehydrated through serial dilutions of ethanol (25%, 50%, 75%, 95%, and 100% each for 10 min, with the 100% twice). The samples were then dried in a critical point dryer (BALTEC, Balzers, Liechtenstein) and mounted on SEM stubs using carbon adhesive tabs. Samples were then sputter-coated with a 10 nm thick layer of gold (BALTEC) and examined with a scanning electron microscope (JSM-5600; JEOL, Tokyo, Japan).

### Statistical analysis

Data are expressed as mean±standard deviation (SD). SPSS software (V:18.0; SPSS Inc., Chicago, IL) was used for statistical analyses. The p value was determined using the Mann–Whitney U test and the Kruskal–Wallis test, as appropriate. Data were considered statistically significant when p<0.05.

## Results

Of the 18 mice in group A and 17 mice in group B, three mice from each group at each time point were euthanized. One mouse from each group died one day after the cryoinjury, and one mouse in group B died 3 days after cryoinjury. Finally, at 21 days after cryoinjury, five mice in group A and three mice in group B remained.

### Central corneal thickness

**Figure 2:**
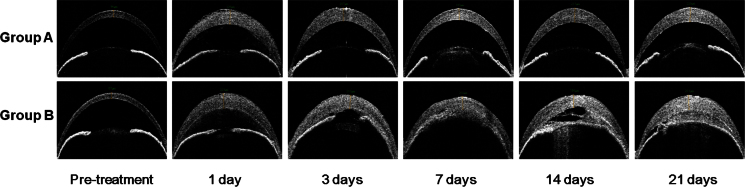
Anterior segment optical coherence tomography. Significant corneal edema developed in groups A and B, and the increased corneal thickness was maintained during the study period. Although the anterior segment appears normal except the corneal edema and mild cataract in group A, severe anterior chamber inflammation and iridocorneal adhesion are remarkable in group B.

In both groups, AS-OCT demonstrated that significant corneal edema developed at day 1 after the cryoinjury, and the edema was sustained during the study period of 21 days (Figure 2). The mean CCT at day 1 was significantly higher than the baseline value in both groups (p<0.001 for both groups A and B, Mann–Whitney U test). During the post-cryoinjury follow-up, no significant differences in the CCT were detected in either group at any time point (p=0.390 for group A and 0.615 for group B, Kruskal–Wallis test), indicating that there had been no significant change in the CCT during the study period. When the two groups were compared, no significant difference in the CCT was detected at any time point (Table 1).

**Table 1:** Comparison of the central corneal thickness (CCT (in μm)) between the two groups (group A: one cycle of cryoinjury, group B: three cycles of cryoinjury)*

Time point	Group A	Group B	P value†
Before Cryo	93.07±7.40 (n=18)	91.81±6.72 (n=17)	0.613
At day 1	194.06±37.28 (n=17)	191.38±36.28 (n=16)	0.836
At day 3	186.88±30.61 (n=14)	183.62±40.77 (n=13)	0.815
At day 7	184.48±20.56 (n=11)	171.19±40.80 (n=9)	0.375
At day 14	168.71±10.83 (n=8)	170.17±23.89 (n=6)	0.880
At day 21	173.67±35.60 (n=5)	170.67±26.66 (n=3)	0.905

*All data are expressed as mean ± SD †Mann–Whitney U test

### Intraocular pressure

Before the cryoinjury, there was no significant difference in the mean IOP between the two groups (8.9±2.0 mmHg for group A and 9.3±1.5 mmHg for group B, p=0.680). However, the mean IOP was higher in group B than that in group A at all time points after the cryoinjury, and the differences were significant at every time point except at day 3 (Table 2). In both groups, the mean IOP increased with time, and there was a significant difference in the mean IOP among the six time points measured during the study period (p=0.003 for group A and 0.001 for group B, Kruskal–Wallis test). In group A, the mean IOP was significantly higher at day 3, 7, and 14 (p=0.040, 0.041 and 0.007, respectively, Mann–Whitney U test) compared to the baseline value, while significantly increased IOP was detected at day 3, 14, and 21 (p=0.011, 0.002 and 0.004, respectively, Mann–Whitney U test) compared to the baseline value in group B. However, the mean IOP remained within the normal IOP range of C57BL/6 mice (10–22 mmHg) in group A, whereas the IOP rose beyond the normal range from day 7 in group B (Table 2) [20,21],

**Table 2:** Comparison of the intraocular pressure (IOP (in mmHg)) between the two groups*

Time point	Group A	Group B	P value†
Before Cryo	8.9±2.0 (n=18)	9.3±1.5 (n=17)	0.680
At day 1	8.3±1.6 (n=17)	11.3±3.3 (n=16)	0.011
At day 3	10.7±2.3 (n=14)	15.1±8.2 (n=13)	0.058
At day 7	12.8±5.3 (n=11)	24.6±16.5 (n=9)	0.038
At day 14	14.8±5.4 (n=8)	29.9±15.8 (n=6)	0.026
At day 21	15.5±8.3 (n=5)	35.3±2.5 (n=3)	0.008

*All data are expressed as mean ± SD †Mann–Whitney U test

### Post-cryoinjury complications

**Figure 3:**
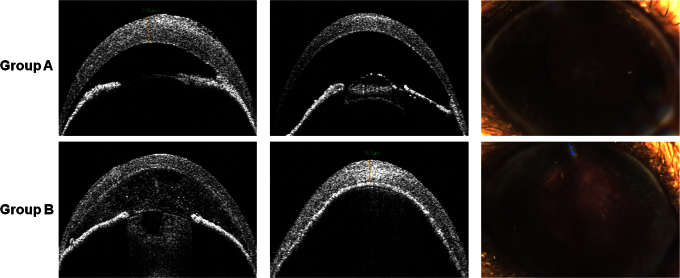
Representative photos showing complications following the cryoinjury. In group A, mild peripheral iridocorneal adhesion (left) and mild cataract (middle and right) were found. In group B, serious complications including severe anterior chamber inflammation and cataract (left), total iridocorneal adhesion (middle), and hyphema (right) were detected.

In group A, mild peripheral iridocorneal adhesion occurred in four cases (from day 7 in two eyes and day 14 in two eyes). Mild cataract was noted in three cases (from day 7 in one eye and day 14 in two eyes). The eye that developed a cataract at day 7 resolved by day 14, but the remaining two cases were irreversible. In group B, severe AC inflammation was detected with AS-OCT in all mice at day 1, and hyphema was found in one case at day 7. Severe iridocorneal adhesion and resultant IOP increase were observed in 15 mice 3 days post-treatment. Development of irreversible cataract was found in all cases, of which three cases were total white cataract (Figure 3).

### Histological examination

**Figure 4:**
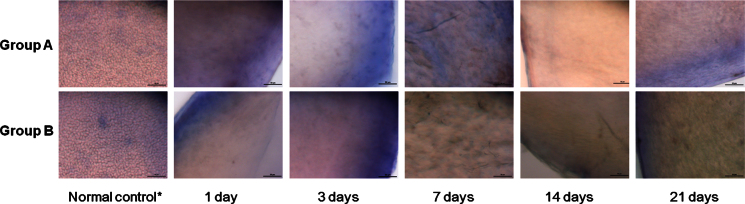
Live/dead cell assay using alizarin red S and Trypan Blue staining of the corneal endothelial cells. Successful ablation of the corneal endothelial cells was observed in samples from day 1, and no evidence of cell regrowth was detected during the study period. Descemet’s membrane was preserved over most of the area. Scale bar=50 μm. *taken from the opposite eye that did not undergo cryoinjury.

In both groups, staining of CECs with alizarin red S and trypan blue showed complete ablation of the corneal endothelial layer and only small clusters of remnant CECs were noted in the periphery (Figure 4). There was no evidence of repopulation of CECs with time noted in either group. In samples from all the time points, DM was mostly preserved, although defects were found in some areas. There appeared to be no significant difference in the extent of DM damage between the two groups and among the various time points.

**Figure 5:**
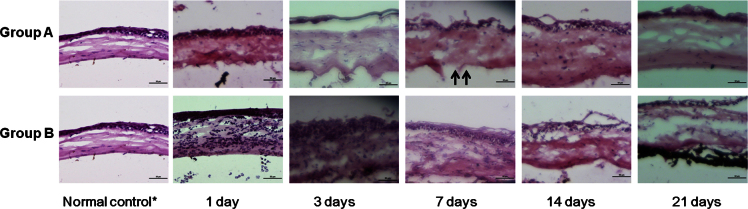
Hematoxylin and eosin staining. Ablation of endothelial cells was observed at all time points in both groups. At 1 day after cryoinjury, more severe infiltration of inflammatory cells throughout all layers of the cornea in group B compared to that in group A was observed. Although Descemet’s membrane (DM) was mostly preserved, detachment of DM was detected in some areas (black arrow). Scale bar=50 μm. *taken from the opposite eye that did not undergo cryoinjury.

H&E staining confirmed the destruction of CECs from both groups at all time points. Although DM remained attached, small focal areas of DM detachment were observed. Immediately after cryoinjury (at day 1), marked infiltration of inflammatory cells was observed throughout all layers of the cornea in group B, whereas there was mild cellular infiltration mainly in the posterior stroma in group A (Figure 5). The inflammation improved with time in both groups, although the corneas in group B appeared to show more severe inflammatory reaction at day 3 and 7 compared to those in group A (Figure 5).

**Figure 6:**
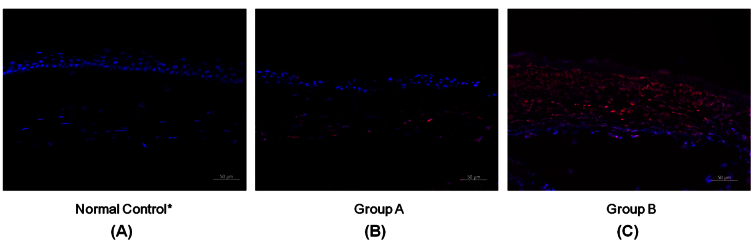
Results of the terminal deoxynucleotidyl transferase-mediated nick end labeling assay. **A**: Apoptosis (red color) was observed only in a few cells in the normal cornea. **B**: In a cornea from group A, apoptosis was detected mainly in the posterior stroma and endothelial layer. **C**: In a cornea from group B, severe apoptosis throughout all layers of the cornea occurred. Scale bar=50 μm. *taken from the opposite eye that did not undergo cryoinjury.

TUNEL assay of the samples taken one day after cryoinjury showed severe apoptosis throughout all corneal layers in group B, whereas apoptosis was mainly detected in the posterior stroma and endothelial layer in group A (Figure 6). The density of apoptotic cells in the cornea from group B was substantially higher than that in the cornea from group A, and the difference was statistically significant (433±168 cells/mm^2^ in group A versus 3167±798 cells/mm^2^ in group B, p<0.001).

H&E staining and TUNEL showed that the corneal epithelial layer was intact at 1 day after the cryoinjury.

### Scanning electron microscopy

**Figure 7:**
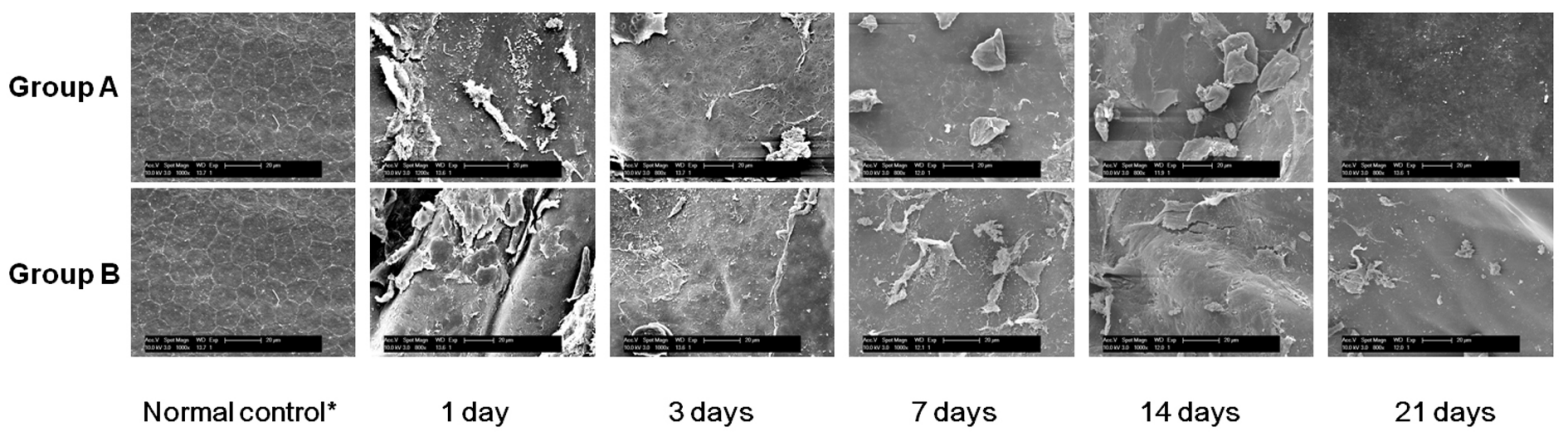
Scanning electron microscopy. At day 1, some corneal endothelial cells (CECs) are still attached to Descemet’s membrane (DM) in both groups, although most of the cells were ablated in both groups. Most of the remnant cells have detached between day 3 and day 14 in both groups. At day 21, denuded DM with only scant distribution of remnant CECs was noted in both groups. Scale bar=20 μm. *taken from the opposite eye that did not undergo cryoinjury.

SEM pictures taken at day 1 showed that low numbers of CECs were still attached to DM in both groups, although most of the cells were removed. Serial SEM showed that remnant cells had been mostly detached from day 3 to 14 in both groups. At day 21, denuded DM with only a scant distribution of small islands of remnant CECs in the periphery were noted in both groups. During the study period, no sign of endothelial cell regeneration (mitosis) was detected (Figure 7).

## Discussion

In the present study, we compared the efficacy and safety between one and three cycles of transcorneal freezing in inducing bullous keratopathy in mice. The results showed that both were equally effective. However, in terms of safety, one cycle of transcorneal freezing appeared to be safer than three.

In both groups, the CCT increased by twofold, and the increased corneal thickness was maintained throughout the study period of 21 days (Table 1 and Figure 2). These findings suggest that even one cycle of transcorneal freezing can effectively induce corneal endothelial decompensation in mouse eyes with minimal complication. The development of the bullous keratopathy was stable for 21 days, which is significantly better than in other animal models, for example, rabbit and rat in which CEC repopulation takes place after only 2 weeks [9,10,22].

The increase in the mean IOP in group B was most likely due to the severe iridocorneal adhesion following cryotherapy. This was associated with serious complications, such as severe AC inflammation (Figure 2 and Figure 3), hyphema, and irreversible cataract formation (Figure 3). In group A, we noted only mild iridocorneal adhesion and mild cataract. The IOP increase even though significantly increased compared to baseline was within the normal range for this mouse species [20,21].

Hematoxylin and eosin staining (Figure 5) and TUNEL assay (Figure 6) also showed substantially more inflammation and apoptosis immediately after the cryoinjury in group B compared to group A, suggesting that three cycles of transcorneal freezing might cause excessive damage to the cornea and adjacent tissues, and one cycle may be sufficient for ablation of the mouse corneal endothelial layer without causing serious complications. The reason may be as follows:1) The corneal thickness (approximately 100 μm) and AC volume (5–6 μL) of mice are substantially smaller than those of other animals, including rabbits, rats, and cats; thus, a decreased dose of transcorneal freezing is enough to destroy CECs [23]. 2) The diameter of the cryoprobe (2.5 mm) is approximately the same size as that of the mouse cornea; thus, the entire CEC layer can be covered with a single application of transcorneal freezing.

The H&E staining, live/dead cell assay, and SEM results revealed that the CEC decompensation was maintained and regeneration of the CECs was not detected during the study period of 21 days (Figure 4, Figure 5, and Figure 7), although a few remnant endothelial cells in the periphery were observed. Although a few remaining CECs in the peripheral area were observed in SEM photos and on live/dead cell assays, no sign of CEC regeneration was detected in any sample (Figure 4 and Figure 7).

The laboratory mouse model is superior to other animals for investigating human disease, due to the availability of transgenic mouse species and various disease models [24,25]. Cryoinjury is a simple extraocular procedure for inducing bullous keratopathy. However, although transcorneal freezing has already been widely used to induce corneal endothelial decompensation in other animal models [1,9-16], use in a mouse model has never been reported, probably because the diameter of the cryoprobe used for other animals (8 mm) is larger than the mouse eye and the dose of cryotherapy; usually two to three shots to the center and several peripheral points, respectively, might deliver excessive damage. Although other methods, including mechanical scraping or injection of toxic agents, have been used for mouse eyes, these intraocular procedures are time-consuming, invasive, and often challenging due to the small size of the eye [7,17]. To overcome such drawbacks, we developed a new mouse model by optimizing the dose of cryoinjury (one cycle) and the diameter of the cryoprobe (2.5 mm). To the best of our knowledge, the present study is the first that demonstrates that transcorneal freezing can be used as an effective and safe method in inducing corneal endothelial decompensation in mouse eyes. We expect that our mouse cryoinjury model will allow further research on possible rescue therapies, such as cell injection therapy or gene transfer using nanoparticles in mice [26,27].

The present study has limitation as follows. 1) The sample size was small, and only one and three cycles of transcorneal freezing were attempted. However, the results consistently showed one cycle of transcorneal freezing is safe and efficient enough for inducing corneal endothelial decompensation in a mouse eye. Thus, the scientific value of subjecting more mice to cryotherapy appears of little utility from the results we have presented. 2) Only a flat-tipped cryoprobe with a diameter of 2.5 mm was used. However, since the C57BL/6 mouse corneal diameter is 2.6 mm, this cryoprobe size is conceivably optimal for cryofreezing on the mouse cornea. A larger probe would cause more severe damage to adjacent tissues, including the trabecular meshwork and lens, and a smaller one would make a smaller area of endothelial cell damage, which would be unsuitable for further endothelial cell rescue therapy. Moreover, the lateral surface of a flat tip has a similar contact surface area to the cornea with the round tip. Therefore, application of the cryoprobe as the lateral surface of the probe contacts the corneal surface would provide similar contact to the cornea surface. 3) As the mouse cornea has a steep curvature compared to the rabbit or monkey, it is difficult to attach the cryoprobe to the peripheral area, which might be associated with the remnant CECs in the periphery. However, the lack of evidence of the regeneration of the cells suggests that our model may be useful for further research on endothelial cell rescue therapy.

In conclusion, one cycle of cryoinjury using a cryoprobe with a 2.5 mm diameter may be effective and safe in inducing disruption of the corneal endothelial cell layer in the mouse eye, whereas three cycles resulted in the same disruption but with excessive damage to ocular tissue. Cryoinjury is an easy, simple, and non-invasive method that can provide an experimental mouse model of bullous keratopathy. Thus, this method is expected to be useful in further research on corneal endothelial decompensation and its treatment.
